# Genome-Wide Association Study Identifies Major Loci for Carcass Weight on BTA14 in Hanwoo (Korean Cattle)

**DOI:** 10.1371/journal.pone.0074677

**Published:** 2013-10-07

**Authors:** Seung Hwan Lee, Bong Hwan Choi, Dajeong Lim, Cedric Gondro, Young Min Cho, Chang Gwon Dang, Aditi Sharma, Gul Won Jang, Kyung Tai Lee, Duhak Yoon, Hak Kyo Lee, Seong Heum Yeon, Boh Suk Yang, Hee Seol Kang, Seong Koo Hong

**Affiliations:** 1 Hanwoo Experiment Station, National Institute of Animal Science, RDA, Pyeongchang, Korea; 2 Animal Genome & Bioinformatics Division, National Institute of Animal Science, RDA, Suwon, Korea; 3 School of Rural and Environment Science, University of New England, Armidale, NSW, Australia; 4 Department of Animal Science, Kyungpook National University, Sangju, Korea; 5 Department of Animal Science, Hankyong National University, Anseong, Korea; University of Sydney, Australia

## Abstract

This genome-wide association study (GWAS) was conducted to identify major loci that are significantly associated with carcass weight, and their effects, in order to provide increased understanding of the genetic architecture of carcass weight in Hanwoo. This genome-wide association study identified one major chromosome region ranging from 23 Mb to 25 Mb on chromosome 14 as being associated with carcass weight in Hanwoo. Significant Bonferroni-corrected genome-wide associations (*P<1.52*×*10^−6^*) were detected for 6 Single Nucleotide Polymorphic (SNP) loci for carcass weight on chromosome 14. The most significant SNP was *BTB-01280026* (*P = 4.02×10^−11^*), located in the 25 Mb region on Bos taurus autosome 14 (BTA14). The other 5 significant SNPs were *Hapmap27934-BTC-065223* (*P = 4.04×10^−11^*) in 25.2 Mb, *BTB-01143580* (*P = 6.35×10^−11^*) in 24.3 Mb, *Hapmap30932-BTC-011225* (*P = 5.92×10^−10^*) in 24.8 Mb, *Hapmap27112-BTC-063342* (*P = 5.18×10^−9^*) in 25.4 Mb, and *Hapmap24414-BTC-073009* (*P = 7.38×10^−8^*) in 25.4 Mb, all on BTA 14. One SNP (*BTB-01143580; P = 6.35×10^−11^*) lies independently from the other 5 SNPs. The 5 SNPs that lie together showed a large Linkage disequilibrium (LD) block (block size of 553 kb) with LD coefficients ranging from 0.53 to 0.89 within the block. The most significant SNPs accounted for 6.73% to 10.55% of additive genetic variance, which is quite a large proportion of the total additive genetic variance. The most significant SNP (*BTB-01280026; P = 4.02×10^−11^*) had 16.96 kg of allele substitution effect, and the second most significant SNP (*Hapmap27934-BTC-065223; P = 4.04×10^−11^*) had 18.06 kg of effect on carcass weight, which correspond to 44% and 47%, respectively, of the phenotypic standard deviation for carcass weight in Hanwoo cattle. Our results demonstrated that carcass weight was affected by a major Quantitative Trait Locus (QTL) with a large effect and by many SNPs with small effects that are normally distributed.

## Introduction

Korean beef producers select for meat yield and quality to increase their income from steer feedlots and sold calves. Estimated breeding values (EBVs) for eye muscle area (EMA), carcass weight (CWT), marbling score (MAR), and backfat thickness (BF) after adjustment for age (30 months) are commonly used as selection criteria in attempts to increase meat yield and quality, which determine profitability for the Korean beef industry. However, current gain-based long-term programs targeting improvement of meat yield and quality in the Korean finishing farm sector have resulted in inefficient meat production because the animals produce excessive fat (subcutaneous and visceral fat). More targeted meat production, such as improved genetic gain for EMA and CWT and more desirable MAR, needs to be achieved in Hanwoo beef industry by genetic improvement. To this end, investigations have been made for incorporation of DNA information into genetic evaluation models, which may result in improved accuracy of estimated breeding value (EBV) and lead to increased selection response in cattle [1]. Finding causal genes having large effects on quantitative traits has the potential to improve animal selection efficiency and increase understanding of the underlying biology of quantitative traits [2].

The recently established genome-wide SNP panel for cattle enables the mapping of quantitative trait loci (QTL) and the prediction of animal's genetic merit, without using phenotypic and pedigree records (Goddard and Hayes, 2009). This approach has been used for fine mapping of QTL for milk traits in dairy cattle [3]–[5]. In beef cattle, whole genome association studies with SNP arrays have identified a number of QTLs for feed intake traits [6]–[8]. As for the bovine stature and growth traits, two studies have revealed a major QTL for bovine stature on chromosome 14 [8]–[9]. Karim et al. (2011) reported a QTL with a major effect on bovine stature at a 780 kb interval on bovine chromosome 14, resulting in two candidate QTL being mapped to the *PLAG1-CHCHD7*. Nishimura et al. (2012) revealed three major QTLs for carcass weight on chromosomes 6, 8, and 14, including the *PLAG1-CHCHD7* QTL for stature in Japanese Black cattle. Most of the agriculturally important traits are complex in their genetic architecture due to their polygenic nature as many genes have small effects and it makes it difficult to identify and characterize individual QTLs for genetic prediction [10]–[11]. A genome-wide association study can significantly aid our understanding of genetic architecture for complex traits. Hayes et al. (2010) reported that genome-wide associations revealed the number of loci affecting coat color and milk-fat percentage and the distribution of their effects, in terms of the accuracy of breeding value predictions.

An important prerequisite for unbiased QTL mapping is homogeneity of the mapping population [12]. A feature of the recent Hanwoo (Korean cattle) population is that is has a small effective population (n = 98) as a result of AI using a small number of sires, and because of this, nonzero (*r^2^* = 0.1) levels of LD have been found in Hanwoo of up to 100 kb [13]. Because the genome-wide association study (GWAS) relies on the LD between SNP markers and QTL for the traits in cattle, the GWAS's using a BovineSNP50 BeadChip positioned at every 100 kb should be adequate to identify significant SNPs associated with carcass traits in Hanwoo. The aim of the current study is to identify the major QTLs affecting carcass traits and the distributions of SNP effects for the traits using the whole genome association study in Hanwoo (Korean cattle).

## Materials and Methods

### Ethics statement

No ethics statement was required for the collection of DNA samples. DNA was extracted either from AI bull semen straws or from blood samples obtained from different veterinary practitioners in the Hanwoo Improvement Center of the National Agricultural Cooperative Federation with the permission of the owners. Both the semen and the blood samples were collected for routine veterinary procedures and not explicitly for the purpose of this study.

### Animals and traits

Data were obtained from 1,011 Hanwoo steers derived from 118 sires from progeny testing in the Hanwoo Improvement Center of the National Agricultural Cooperative Federation in Seosan, Chungnam province, Korea. The sample comprised paternal half-sib pedigrees from 118 proven Korean sires. The steers were born from spring of 2005 through fall of 2007 and weaned at 5 or 6 months of age, and each group of 10 steers were raised in a pen. The steers received an *ad libitum* ration of a total mixed diet of concentrates and rice straw with ratios in total feed of about 1.5∶1, 2∶1, and 4.5∶1 for the growing period (4–12 months), finishing period I (13–18 months), and finishing period II (19–23 months), respectively. Percentage of crude protein (CP) and total digestible nutrients (TDN) of the concentrates were, respectively, 14–16% and 68–70% for the growing period, 11–13% and 71–73% for finishing period I, and 11% and 72–73% for finishing period II. Phenotype data in this study included carcass weight (CWT), eye muscle area (EMA), backfat thickness (BF), and marbling score (MAR). BF, EMA, and MAR were measured at the 12^th^ and 13^th^ rib junction after a 24-hour chill. MAR was assessed on a one-to-nine point scale, and the degree-of-marbling score was evaluated based on the Korean Beef Marbling Standard from the Animal Product Grading Service in Korea [14]. Statistics for the phenotype data used in this study are summarized in Table 1.

**Table 1:** Trait means, standard deviations, variance components for the carcass traits, and number of animals in the Hanwoo discovery set.

Traits	Phenotypic Data				Variance components		
	Mean	SD	Min	Max	σ^2^_g_	σ^2^_p_	h^2^
**Carcass weight (kg)**	356.4	37.8	183	488	347.6	1056.1	0.33
**Eye muscle area (Cm^2^)**	81.1	8.6	59	111	23.7	57.6	0.41
**Marbling score (1–9)**	3.1	1.5	1	9	1.43	2.86	0.50
**Backfat thickness (mm)**	8.9	3.6	2	35	4.5	11.2	0.40

### Genotype assay

Genomic DNA for genotyping assays was extracted from the blood sample, and the DNA was isolated from the blood using DNeasy 96 Blood and Tissue Kit (Qiagen, Valencia, CA, USA). DNA quantification was performed using a NanoDrop 1000 (Thermo Fisher Scientific Inc., Wilmington, DE, USA). DNA samples were submitted for genotyping with total DNA of 900 ng, 260/280 ratio >1.8, and DNA concentration of 20 ng/ul.

The genotyping for 1,020 animals was done by the Animal Genome & Bioinformatic Division of the National Institute of Animal Science, RDA, Korea, using a BovineSNP50 BeadChip Ver. 1 and Bovine HD 777 K Chip (Illumina, San Diego, CA, USA, [15]). Nine animals were removed in 50 K SNP data set because of missing genotypes (>50%). In order to increase sample size, we matched common SNPs from 50 K (n = 803) and 777 K SNP chip (n = 288) which resulted in 43,439 SNPs. Criteria for selecting SNPs were, 80% call rate, MAF >0.001 and Hardy Weinberg <1E-5. Many of SNPs (n = 3591) were not polymorphic in Hanwoo. 342 SNPs were with 20% missing genotypes, 1583 SNPs had MAF <0.001 was and finally 5227 SNPs had HWE <1E-5 in Hanwoo. 32,696 SNPs met all of these criteria. Of these 32,696 SNPs, unmapped SNPs (n = 835) and sex chromosome (n = 44) were not plotted in Manhattan plot.

The BovineSNP50 BeadChip Ver 1 (Illumina, San Diego, CA, [15]) was used to genotype 1,011 steers from a progeny-tested population. To test the associations between SNPs and the QTL, a single-marker linear mixed model was implemented using ASREML [16]. Markers were assumed to be in LD with QTL in close proximity, and the effect was evaluated to be an additive effect (QTL allele substitution effect). The additive effect was fitted to a regression of phenotype on allele counts (0, 1, and 2). The additive effect of a SNP on each phenotype was calculated by regression analysis, with values in the covariate coded as 0, 1, or 2 copies of the variant allele, and after fitting the following mixed-model:
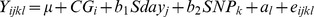
where 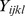 is a vector of carcass traits, 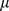 is a vector of overall mean for carcass traits, 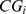 is a vector of contemporary group for birth year, season and batch effects, 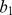 is a regression coefficient, 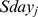 is a vector of the day of slaughter as a covariate effect, 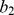 is the marker genotype effect, 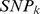 is a single-locus SNP genotype coded as 0, 1 or 2 as a covariate effect, 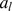 is a vector of random polygenic effect ∼**N** (**0,** 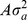) and e is a vector of random residual ∼**N** (**0,** 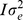).

Quantile-quantile plots were investigated to judge the extent of false positive signals. SNPs were considered as significantly associated for P-values below the 5% Bonferroni-corrected type-I error threshold for 32,696 independent statistical tests. Allele substitution effects were estimated for each significant marker in a linear regression model implemented in ASREML [16]. Two SNPs were selected for haplotype association analysis. For the two selected SNPs, we reconstructed haplotypes across all these loci using the PHASE program [17]. Haplotype effects were fitted as separate effects in the model, with the haplotype count fitted as a covariate.

The percentage of the genetic variance explained by each significant SNP was calculated. The percentage of the genetic variance accounted for by the *i*-th SNP was computed according to the formula:
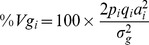
where 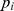 and 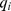 are the allele frequencies for the *i-th* SNP estimated for Hanwoo, 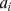 is the estimated additive effect of the *i-th* SNP on the phenotype in equation, and 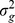 is the REML estimate of the polygenic variance for carcass traits in Hanwoo.

To investigate linkage disequilibrium (LD) and allele frequencies for six significant SNPs among breeds, five European cattle breeds (Angus, Brahman, Limousine, Hereford, and Holstein) and five Northeast Asian cattle breeds (Korean brown Hanwoo, Korean brindle Hanwoo, Jeju black Hanwoo, Chosun cattle, and Yanbian cattle) were used. The 6 SNPs' data for the 10 breeds were selected from the bovineSNP50 BeadChip data. The sample size for the 10 breeds was 20 animals for each. The haplotypes for the 6 significant SNPs in the 25 Mb region on BTA14 were reconstructed using default parameters in PHASE [17] and inspected by means of Heatmap plots obtained with Haploview [18] to visualize recombination events and to define the length of haplotypes.

The STRUCTURE software was applied to study genetic structure, the degree of population uniformity, and admixture in the chosen cattle breeds [19]. The program implements a model-based clustering method for inferring population structure using genotype data. There were 20,000 burn-in steps before 50,000 MCMC repeats. The number of clusters (*K*) ranged from 2 to 14, with 10 repeat runs for each *K* value under the admixture model.

To annotate SNPs with respect to their functional classification on genes, we downloaded bovine gene annotation data sets (gene annotations and FASTA sequences) from the UCSC Genome Browser. The SNPs were annotated using the ANNOVAR tool (version 2012) [20]. For region-based annotation of SNPs, the ANNOVAR annotates the location of each variant with respect to genes, indicating whether it is exonic, intronic, intergenic, a splicing site, 5′/3′-UTR, and upstream/downstream of genes.

## Results

In this genome-wide association study, the 1,011 steers descended from 118 sires were used as a discovery data set to identify the QTL for carcass traits (CWT, EMA, MAR, and BF). The paternal half-sib families had up to 118 members. The wide-spread use of a limited number of sires in Hanwoo cattle results in extensive linkage disequilibrium (LD), however it also causes a subtle form of admixture due to relationships among the animals. If unaccounted for, the relationship can lead to a large number of false positives in the GWAS. The carcass traits of the samples had divergent phenotypes; for example, carcass weights ranged from 183 kg to 488 kg (Table 1) but were normally distributed. The 32,696 SNPs on autosomes and sex chromosome that fulfilled the SNP quality control criteria (MAF<0.001 and HWE<1E-5) were used for the association analysis. The GWAS analysis was performed using a linear mixed-model approach that employed a numeric relationship matrix estimated based on the animals' pedigrees.

**Figure 1:**
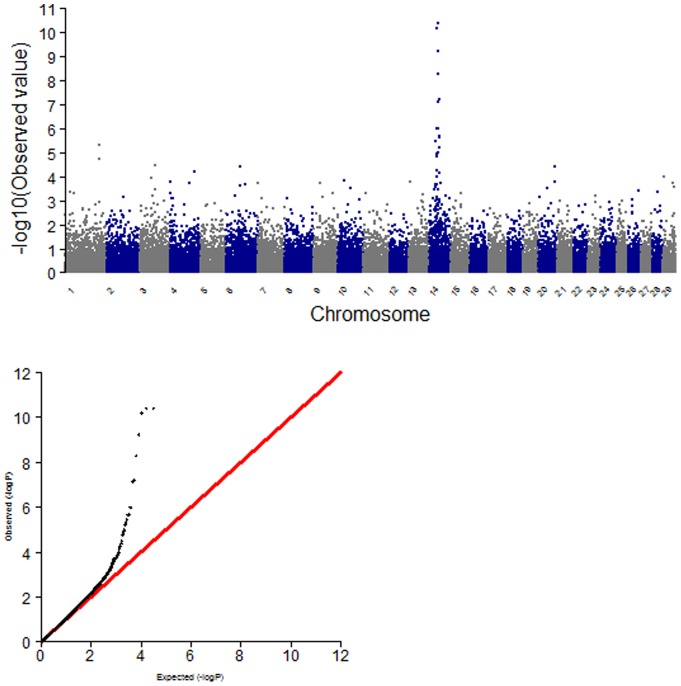
Association of 32,696 SNPs with the carcass weight in the Hanwoo breed. (A) Manhattan plot. Significance threshold was set up at P<1.5×10^−6^ (Bonferroni corrected significance level). (B) Quantile-quantile plot. The red line represents the 95% concentration band under the null hypothesis of no association. The black dot represent the P-values of the entire study, upper six dots represent SNPs with P<1×10^−8^ in this study.

The GWAS identified one major chromosome region ranging from 23 Mb to 25 Mb on chromosome 14, which is associated with carcass weight in Hanwoo (Figure 1). The quantile-quantile (Q-Q) plot with P<10^−3^ for carcass weight revealed a large deviation from the distribution under the null hypothesis, indicating that strong associations were obtained (Figure 1). Significant Bonferroni-corrected genome-wide associations (*P<1.52*×*10^−6^*) were detected for 6 SNPs for carcass weight on chromosome 14 (Figure 1). The most significant SNP was *BTB-01280026* (*P = 4.02*×*10^−11^*) located in the 25 Mb region on BTA14 (Figure 1). The other 5 significant SNPs were *Hapmap27934-BTC-065223* (*P = 4.04*×*10^−11^*) in the 25.2 Mb region, *BTB-01143580* (*P = 6.35*×*10^−11^*) in the 24.3 Mb region, *Hapmap30932-BTC-011225* (*P = 5.92*×*10^−10^*) in the 24.8 Mb region, *Hapmap27112-BTC-063342* (*P = 5.18*×*10^−9^*) in the 25.4 Mb region, and *Hapmap24414-BTC-073009* (*P = 7.38*×*10^−8^*) in the 25.4 Mb region, all on BTA 14.

**Figure 2:**
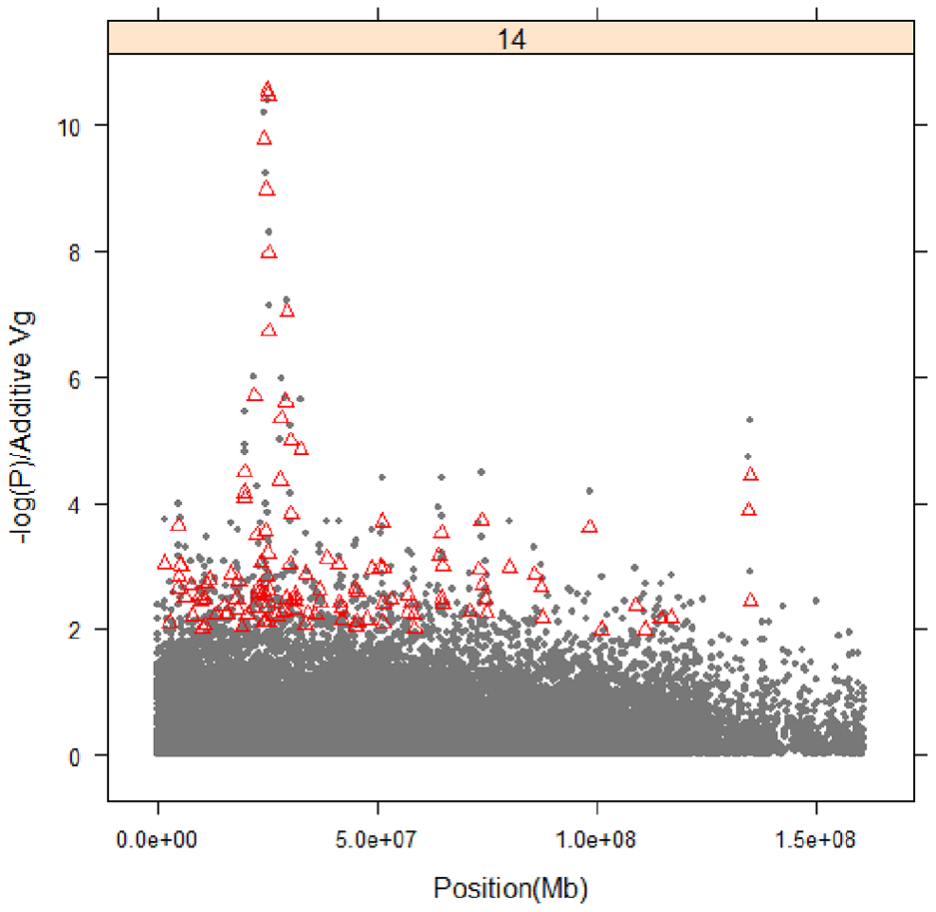
The -logP value for the association between SNP and carcass weight on chromosome 14 (grey colour) and additive genetic variance (red colour) that significant SNPs (P<0.001) account for in single marker regression analysis.

**Figure 3:**
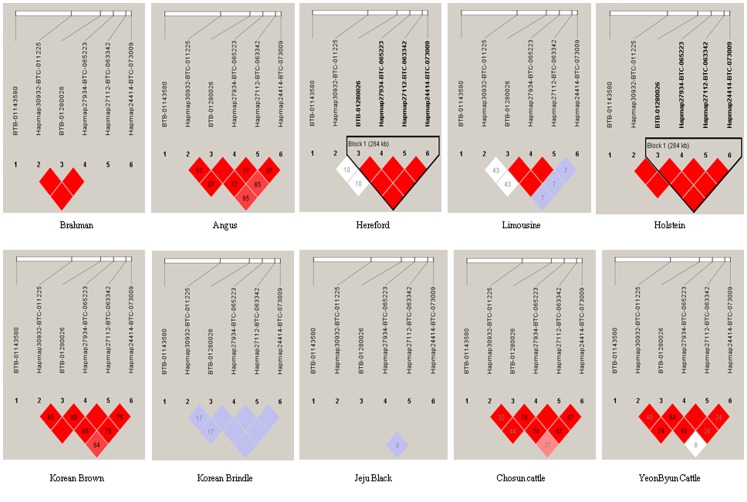
Linkage disequilibrium of the 6 significant SNPs. Linkage disequilibrium pattern between SNPs in the 1.1

We calculated the proportion of additive genetic variance explained by the SNPs, meeting a P-value of 0.001, detected in single-marker regression analysis (Figure 2). As shown in Figure 3, each of the SNPs between the threshold *P = 0.001* and *P = 10^−6^* explained only 2 to 5% of additive genetic variance, which is a quite small proportion of the total additive genetic variance. However, the most significant SNPs accounted for 6.73 to 10.55% of the additive genetic variance, which is quite a large proportion of the total additive genetic variance (Table 2). In addition, the allele substitution effects for each of the significant SNPs were calculated using linear regression analysis (Table 2). The most significant SNP (*BTB-01280026; P = 4.02×10^−11^*) had 16.96 kg of allele substitution effect, and the second most significant SNP (*Hapmap27934-BTC-065223; P = 4.04×10^−11^*) had an effect of 18.06 kg for carcass weight, which corresponds to 44% and 47%, respectively, of the phenotypic standard deviation for carcass weight in Hanwoo (Table 2). As for the GWAS for other traits (EMA, BF, and MAR), no highly associated signals were observed for these traits (Figure S1). CWT is genetically correlated with the EMA in Hanwoo, but the association signal did not meet a Bonferroni-corrected significance threshold (*P<10^−6^*) in this study. In addition, there were no highly associated signals for fat traits such as BF and MAR. For these traits, it is possible that a large number of genes with small effects are scattered across the whole genome in Hanwoo (Figure S2).

**Table 2:** SNPs showing significant association with carcass weight in 1,011 Hanwoo animals.

SNP	Gene	Chr	Position	P-value	n	n0	n1	n2	p	q	Vg	β (kg)
BTB-01143580	FAM110B	14	24383627	6.35×10^−11^	1010	793	200	17	0.88	0.12	9.77	17.31
Hapmap30932-BTC-011225	SDCBP	14	24898781	5.92×10^−10^	1011	792	203	16	0.88	0.12	8.97	16.57
BTB-01280026	TOX	14	25170557	4.02×10^−11^	1004	756	230	18	0.87	0.13	10.55	16.96
Hapmap27934-BTC-065223	TOX	14	25288714	4.04×10^−11^	1009	795	198	16	0.89	0.11	10.46	18.06
Hapmap27112-BTC-063342	TOX	14	25405377	5.18×10^−9^	1011	827	173	11	0.90	0.10	7.97	16.77
Hapmap24414-BTC-073009	TOX	14	25455256	7.38×10^−8^	1011	769	2226	16	0.87	0.13	6.73	13.74

Six SNPs meet the genome wide significance level of *P<1.52×10^-6^*. The allelic substitution effect (β) is given for the minor allele in additive genetic effect derived from linear regression model of the carcass weight (kg). Positions are based on the Btau 4.1 assembly of the bovine genome sequence. The Vg indicates proportion of SNP vairance calculated from equation in material and methods.

**Figure 4:**
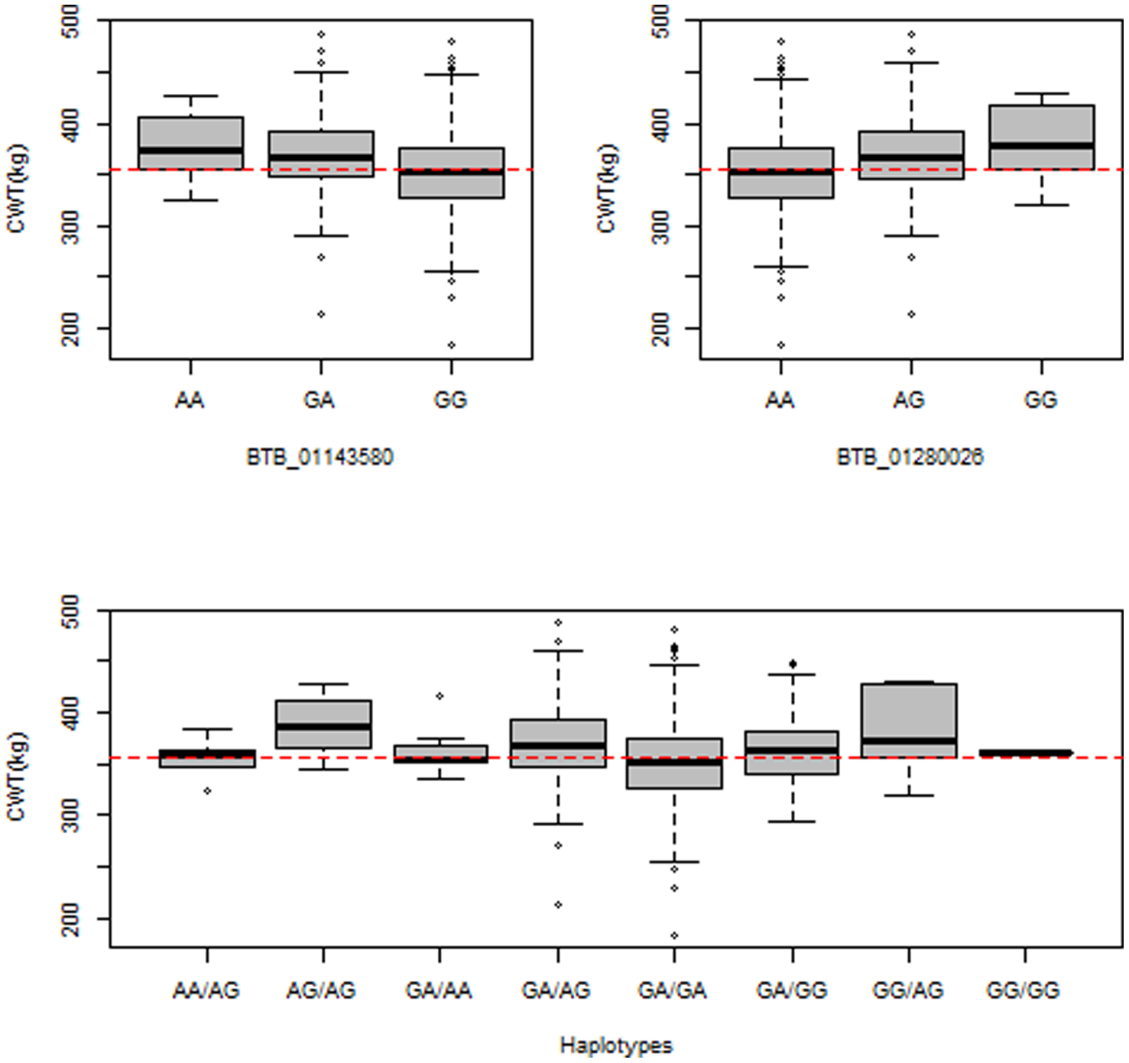
Effect of the most significantly associated SNPs (*BTB_01143580* and *BTB_01280026*) on the carcass weight in the Hanwoo breed. The boxplots show the effects of the most significantly associated two SNPs and their haplotypes on the carcass weight.

Haplotype analysis was performed for the 6 significant SNPs to delineate the chromosome segment carrying the carcass weight QTL. Of the 6 significant SNPs on BTA14, one SNP (*BTB-01143580; P = 6.35×10^−11^*) lies independently from the other 5 SNPs (Figure 3). Therefore, we investigated the haplotype effect of the two SNPs (*BTB-01143580; P = 6.35×10^−11^* and *BTB-01280026; P = 4.02×10^−11^*) that span the 0.6 Mb region (starting at 24.3 Mb; Table 2). For these two SNPs on chromosome 14, the alleles that increase carcass weight by 17.3 kg and 17 kg occur with frequencies of 13% and 14%, respectively, in the study population. The haplotype AG/AG occurs with a frequency of 10% in the study population. Its positive effect on carcass weight (*P = 0.00001*) is more prominent than other two of the associated SNPs (380 kg vs. 360 kg for carcass weight; Figure 4).

We investigated LD structure (*r^2^* value) for the major region of the 1.1 Mb interval (from 24. Mb to 25.4 Mb) which harbored 29 SNPs (Figure S3). Five LD blocks were found with LD coefficients (*r^2^*) ranging from 0.1 to 0.94 within each LD block (Figure S3). The most significant SNP (*BTB-01280026; P = 4.02×10^−11^*) was located in block 5 (block size of 234 kb) along with the other 2 significant SNPs (*Hapmap27934-BTC-065223*; *P = 4.04×10^−11^* and *Hapmap27112-BTC-063342*; *P = 5.18×10^−9^*).

As secondary evidence of SNP effects on carcass weight, linkage disequilibrium and allele frequencies for favorable alleles of the 6 significant SNPs in the 1.1 Mb were compared in five European breeds with large body stature and five Northeast Asian breeds (Korean and Chinese breeds) with relatively small body stature. The favorable allele frequency was different in European (from 25% to 77%) and Northeast Asian (from 0% to 14%) breeds in all SNPs. In Brahman, two SNPs (*Hapmap27112-BTC063342*; 25405377bp and *Hapmap24414-BTC-073009*; 25455256bp) were not polymorphic. This might be because Brahman cattle have a different genetic background as a bos indicus. In this study, we also compared favorable allele frequencies for three Hanwoo breeds (Korean brown, Korean brindle, and Jeju black with different coat colors). Interestingly, the favorable allele frequencies of the 6 SNPs in Korean brown cattle were different from those in Korean brindle and Jeju Black Hanwoo cattle (Table 3). Korean brown Hanwoo had about 11–14% of the favorable allele frequencies, whereas Korean brindle had about 3% of the favorable alleles. In Jeju black, the favorable alleles for 4 SNPs (*BTB-01143580, Hapmap30932-BTC-011225, Hapmap27934-BTC-065223*, and *Hapmap27112-BTC063342*) were not detected, and the other two SNPs (*BTB-01280026* and *Hapmap24414-BTC-073009*) were observed at very low allele frequencies (8% and 5%, respectively). This may be because crossbreeding of brown Hanwoo with European breeds in the past was the source of the favorable allele (Figure 3). The admixture of European with Asian cattle breeds was clearly observed in the structure results (Figure S4). Also, the brown Hanwoo breed has faced high selective pressures for meat yield, which could be another reason for the higher frequency of the favorable allele, compared to brindle and Jeju black.

**Table 3:** Frequencies of favorable alleles of 6 SNP markers for carcass weight in the 1.1

SNP	Position	Allele	FA	AG (n = 20)	BR (n = 20)	HT (n = 19)	HF (n = 15)	LM (n = 15)	KB (n = 1,011)	CHB (n = 20)	YBH (n = 39)	CS (n = 19)	JBB (n = 20)
BTB-01143580	24383627	G: A	A	0.35	0.15	0.32	0.47	0.77	0.13	0.03	0.1	0.13	0
Hapmap30932-BTC-011225	24898781	G: A	A	0.23	0.43	0.26	0.47	0.58	0.13	0.13	0.04	0.11	0
BTB-01280026	25170557	A: G	G	0.3	0.43	0.26	0.2	0.65	0.14	0.03	0.09	0.26	0.08
Hapmap27934-BTC-065223	25288714	A: G	G	0.3	0.43	0.26	0.2	0.65	0.12	0.03	0.05	0.21	0
Hapmap27112-BTC-063342	25405377	G: A	A	0.25	0	0.26	0.2	0.65	0.11	0.03	0.05	0.21	0
Hapmap24414-BTC-073009	25455256	C: A	A	0.28	0	0.26	0.2	0.96	0.14	0.03	0.18	0.32	0.05

FA  =  favorable allele for carcass weight in Hanwoo (Korean brown cattle).

AG  =  Angus, BR  =  Brahman, HT  =  Holstein, HF  =  Hereford, LM  =  Limousine, KB  =  Korean brown Hanwoo, CHB  =  Korean Brindle Hanwoo, YBH  =  Chinese Yanbian cattle, CS  =  Chosun cattle and JBB  =  Jeju Black Hanwoo.

The LD structure with the six SNPs meeting the Bonferroni-corrected threshold for CWT revealed different LD structures among the cattle breeds (Figure 3). One SNP (*BTB-01143580; P = 6.35×10^−11^*) lay independently from the other 5 SNPs in Korean brown, Angus, Holstein, Chosun, and Yanbian cattle (Figure 3). The other 5 SNPs showed a large LD block (block size of 553 kb) with LD coefficients ranging from 0.53 to 0.89 within the block in these breeds (Figure 3). However, the LD structure of the 6 SNPs for Korean brown Hanwoo was quite different from that of two other Korean cattle breeds (brindle and Jeju black).

**Figure 5:**
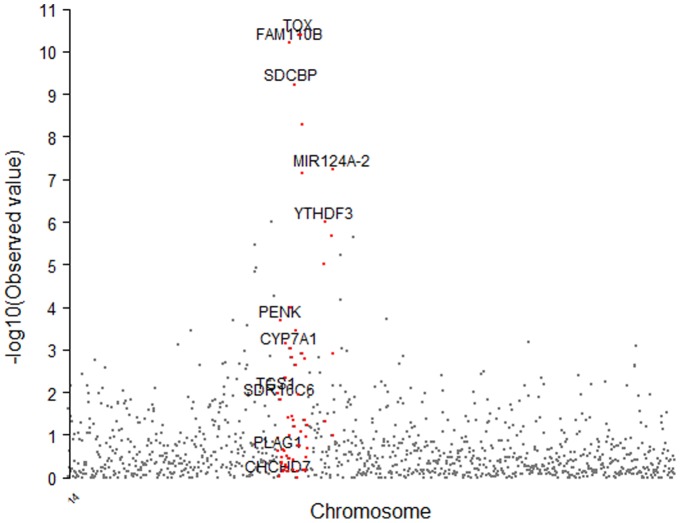
Reginal plot of the p values on chromosome 14 and candidate genes surrounding the six most significant SNPs. Significance (-log 10 of p-values) is plotted against position along the BTA14 and the 1.1 Mb region surrounding the six significant SNPs.

The assessment of the transcriptional content of the carcass weight–associated regions was based on the Btau 4.0 assembly and annotation of the bovine genome. As shown in Table 2, the most significant SNPs for carcass weight were positioned in the 24.4–25.5 Mb region in this study. However, we investigated the broader genome region from 22.8 Mb to 26.4 Mb (3.6 Mb intervals) on chromosome 14. The genome region (22.8–26.4 Mb) encompasses 9 genes located within at least 0.5 Mb (Figure 5). The associated region on bovine chromosome 14 is conserved in human chromosome 8q21, which has been reported to be associated with adult height [21]. We identified *PLAG1, CHCHD7, SDR16C5, SDR16C6, PENK, FAM110B, CYP7A1, SDCBP*, and *TOX* as positional and functional candidate genes for the carcass weight QTL in cattle. However, the genes that are closer to the most significant SNPs are *FAM110B, SCDBP*, and *TOX* in this study. In this GWAS study, we identified the 6 most significant SNPs near *TOX*, *FAM110B*, and *SDCBP* for carcass weight in Hanwoo. This finding does not correspond to previous studies, where *PLAG1* gene was a causal gene for bovine stature, and *RPS20* (*ribosomal protein S20*) gene was for calving ease that is highly correlated with fetal growth traits in cattle. According to long range LD results (Figure S3) a denser LD structure was found in and around TOX gene rather than a region that surrounds PLAG1 gene. This result might be due to a multigene effect in which multiple genes in the same QTL region are affecting correlated traits in cattle.

## Discussion

This genome-wide association study using a dense 50 K SNP chip identified a major QTL for carcass weight on chromosome 14 that explain at least 10% of the genetic variation of carcass weight in Hanwoo cattle. Importantly, the major QTL with large genetic variations for CWT could be useful for marker-assisted selection to determine animal destiny for feedlots or seed stocks in the Korean beef industry. However, the other carcass traits, such as EMA, BF, and MAR, were not detected in any SNP markers that met the Bonferroni-corrected threshold (*P<1.52*×*10^−6^*). In particular, carcass weight is genetically correlated with EMA (r_G_ = 0.45), but no corresponding SNPs achieving the Bonferroni-corrected threshold (*P<1.52*×*10^−6^*) were associated with EMA on chromosome 14, although there is an association signal with EMA in a corresponding region on chromosome 14 (Figure S1). This might be due to insufficient sample size (n = 1,011) to successfully map QTL with small proportions of genetic variation for complex traits. Pausch et al. (2011) [22], calculated the adequate sample size to successfully map QTL for complex traits with very low heritability. He suggested that, if the QTL has a heritability of 0.08, LD between marker SNPs and QTL, i.e., *r^2^* = 0.35 and 4% of the genetic variation, then approximately 20,000 animals would be required for the successful identification of QTL.

Another key factor for mapping QTL is carefully corrected animal relatedness in the study population. In the Hanwoo production system, the use of artificial insemination (AI) from a few elite sires has created not only an extensive LD structure but also a subtle form of admixture of relationships among the animals, which causes LD between SNP markers even if they are unlinked [2]. Such relationships could result in a large number of false positives in the GWAS. Addressing control of such false positives, MacLeod et al. (2006) [23] suggested including a component to account for the relationships in the statistical model based on known pedigrees. Pausch et al. (2011) [22], showed an inflation of significant associations for a statistical model without correction of animal relatedness compared to one with animal relationships in 1800 bulls of the German Fleckvieh breed. In the current study, we used a statistical model with a component of animal relationships to remove animal relatedness and relationships in the discovery population. The major QTL region with at least 10% of genetic variation on BTA14 in Hanwoo also explain a substantial fraction of the calving ease in German Fleckvieh [24], bovine stature (height) in the Holstein-Friesian (HF) × Jersey line [8], and growth related traits in Japanese black [9]. Possibly related, the major QTL region harboring 6 SNPs for CWT on BTA14 in Hanwoo has been identified on correlated traits, such as bovine fetal growth and calving ease in other cattle breeds. Therefore, LD structure and frequencies for favorable alleles of the 6 significant SNPs in the 1.1 Mb were compared in 5 European breeds with large body stature and 5 Northeast Asian breeds (Korean and Chinese breeds) with relatively small body stature. The results revealed a SNP (*BTB-01143580; P = 6.35×10^−11^*) that lay independently from the other 5 SNPs in Korean brown, Angus, Holstein, Chosun, and Yanbian cattle. The other 5 SNPs showed a large LD block (block size of 553 kb) with LD coefficients ranging from 0.53 to 0.89 within the block in these breeds. However, the LD structure of the 6 SNPs for Korean brown Hanwoo was quite different from that of two other Korean cattle breeds (brindle and Jeju black). As for the allele frequencies for the 6 significant SNPs, the favorable allele frequency was about 25 to 77% in European and 0 to 14% in Northeast Asian breeds. Interestingly, the favorable allele frequencies of the 6 SNPs in Korean brown were different from Korean brindle and Jeju black Hanwoo, as were the LD structures. Korean brown Hanwoo had around 11–14% favorable allele frequencies, whereas Korean brindle had about 3%. In Jeju black, the favorable alleles for 4 SNPs (*BTB-01143580, Hapmap30932-BTC-011225, Hapmap27934-BTC-065223*, and *Hapmap27112-BTC063342*) were not detected, and the other two SNPs (*BTB-01280026* and *Hapmap24414-BTC-073009*) were observed with very low allele frequencies (8% and 5%, respectively). Thus, the favorable allele was common (25 to 77%) in European breeds, while they showed much lower frequencies in Northeast Asian cattle. Recent genetic introgression has been performed to improve growth rate and meat yield in the Hanwoo population. A Holstein-Angus-Charolaise sire was used within the Korean brown Hanwoo population from the early 1970s to 1980s to improve growth rate and meat yield [25]–[26]. Given that the favorable allele frequencies exist in Korean brown and brindle cattle, these favorable alleles may have been introduced from this sire into Korean brown Hanwoo. In contrast, the Jeju black Hanwoo breed has been bred on isolated Jeju Island in Korea, there has been no known outcrossing, and so no favorable alleles exist in the Jeju black Hanwoo.

Our finding of major QTL at 24.3–25.4 Mb on BTA14 is supported by several previous studies in cattle. Kneeland et al. (2004) [27], mapped three QTL regions associated with birth weight in a composite breed. The proximal QTL region was 26.7 cM, which corresponds to the highly significant QTL region for carcass weight identified in this study. Koshkoih et al. (2006) [28], provided evidence for birth weight QTL at the 26–50 cM region on chromosome 14 in a cross of Limousin and Jersey cattle. Maltecca et al. (2009) [29], identified a birth weight QTL at the 19 cM region on chromosome 14 in a Jersey-Holstein cross. QTL for growth and CWT were mapped with 1.1 Mb intervals on BTA14 in a purebred Japanese Black (Wagyu) [30]. In Japanese Black cattle, the QTL region for CWT was in a region close to where a QTL for CWT was detected in this study. For Hanwoo cattle, Lee et al. (2011) [31], identified the most likely QTL region as being at 46–53 cM for carcass weight, and 16 candidate genes were detected within the QTL region in their investigation of gene expression of the candidate genes in muscle samples from highly divergent CWT animals.

Recent studies have revealed a major QTL for bovine stature on chromosome 14 [8]–[9]. Karim et al. (2011) [8], reported a QTL with a major effect on bovine stature at a 780 kb interval on bovine chromosome 14, resulting in two candidate QTL mapped to the *PLAG1-CHCHD7*. *Pleomorphic adenoma gene 1 (PLAG1*) and *CHCHD7* as possible causative genes for bovine stature. *PLAG1* gene is known to affect levels of *IGF2* and other growth factors. The two QTL mapped to an intergenic region of *PLAG1-CHCHD7* modulated bidirectional promoter strength and affected binding of nuclear factors. Nishimura et al. (2012) [9], revealed three major QTLs for carcass weight on chromosomes 6, 8, and 14, including the *PLAG1-CHCHD7* QTN for stature in Japanese Black cattle. In addition, Pausch et al. (2011) [22], identified two calving ease–associated QTLs on chromosomes 14 and 21 in the German Fleckvieh (FV) breed. The most significant QTL region on chromosome 14 was the 24.06–25.5 Mb region, based on the UMD 3.1 position (23–24 Mb on Btau4.1 position). Re-sequencing of positional candidate genes within the QTL region on BTA14 identified highly significantly associated polymorphism modulating a polyadenylation signal of the gene encoding *ribosomal protein S20* (*RPS20*). Unlike the QTL for bovine stature and carcass weight reported by Karim et al. (2011) and Nishimura et al. (2012) [8]–[9], our finding revealed the most significant QTL region (25.1 Mb on Btau4.1 position) to be in a region that is 1 Mb away from the QTL region they reported. The major region encompassing the most significant SNPs included three genes (*FAM110B, TOX*, and *SDCBP*). However, the SNPs located close to *PLAG1-CHCHD7* did not show significant associations with CWT. Fortes et al. (2011) [24], identified *TOX* from this genomic region as a key transcription factor responsible for the molecular regulation of puberty in Brahman cattle.

 Improving carcass weight and eye muscle areas along with marbling scores is a major objective of the Hanwoo breeding program. Animals known to carry favorable alleles for the chromosome 14 QTL could be more stringently selected without antagonistically compromising marbling score. Therefore, the small carcass weight of the Hanwoo cattle could be improved by selection on favorable alleles of the major QTL. This study helps to explain the genetic architecture for carcass weight in Hanwoo cattle, and it provides useful information for marker-assisted selection.

## Supporting Information

Figure S1 **Association of 32,696 SNPs with the eye muscle area (EMA), backfat thickness (BF) and marbling score (MAR) in the Hanwoo breed.** Manhattan plot. Significance threshold was set up at P<1.5×10^−6^ (Bonferroni corrected significance level). Quantile-quantile plot. The red line represents the 95% concentration band under the null hypothesis of no association. The black dot represent the P-values of the entire study, upper six dots represent SNPs with P<1×10^−8^ in this study.
(TIF)

Figure S2 **The -logP value for the association between SNP and carcass traits across genome (grey colour) and additive genetic variance (red colour) that significant SNPs (P<0.001) account for in single marker regression analysis.** (A) Eye muscle area, (B) Back fat thickness and (C) Marbling score (MAR).
(TIF)

Figure S3 **Linkage disequilibrium of the 29 SNPs surrounding 1.1** **Mb BTA14 in Hanwoo.**
(PNG)

Figure S4 **STRUCTURE result between Asian and European cattle breeds.**
(TIF)
