# Supplementary figures and images for: Genome-Wide Association Study Identifies Major Loci for Carcass Weight on BTA14 in Hanwoo (Korean Cattle)
Source: PLoS One. 2013 Oct 7;8(10):e74677. doi: 10.1371/journal.pone.0074677 (PMC3792095; doi:10.1371/journal.pone.0074677)

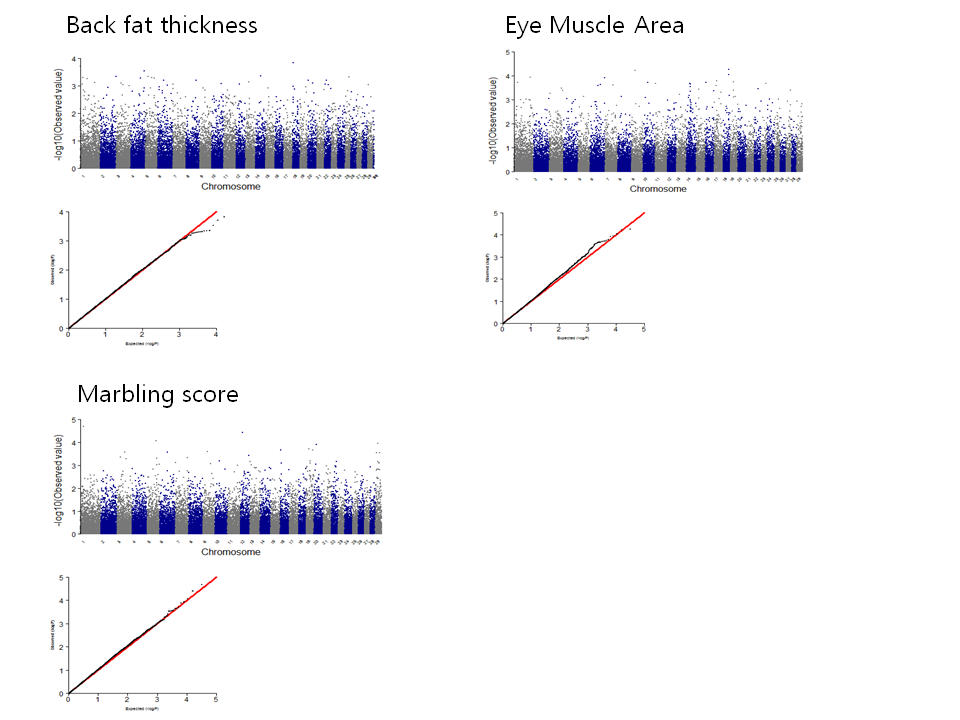

Supplement: Figure S1 — Association of 32,696 SNPs with the eye muscle area (EMA), backfat thickness (BF) and marbling score (MAR) in the Hanwoo breed. Manhattan plot. Significance threshold was set up at P<1.5×10^−6^ (Bonferroni corrected significance level). Quantile-quantile plot. The red line represents the 95% concentration band under the null hypothesis of no association. The black dot represent the P-values of the entire study, upper six dots represent SNPs with P<1×10^−8^ in this study. (TIF) [file pone.0074677.s001.tif]

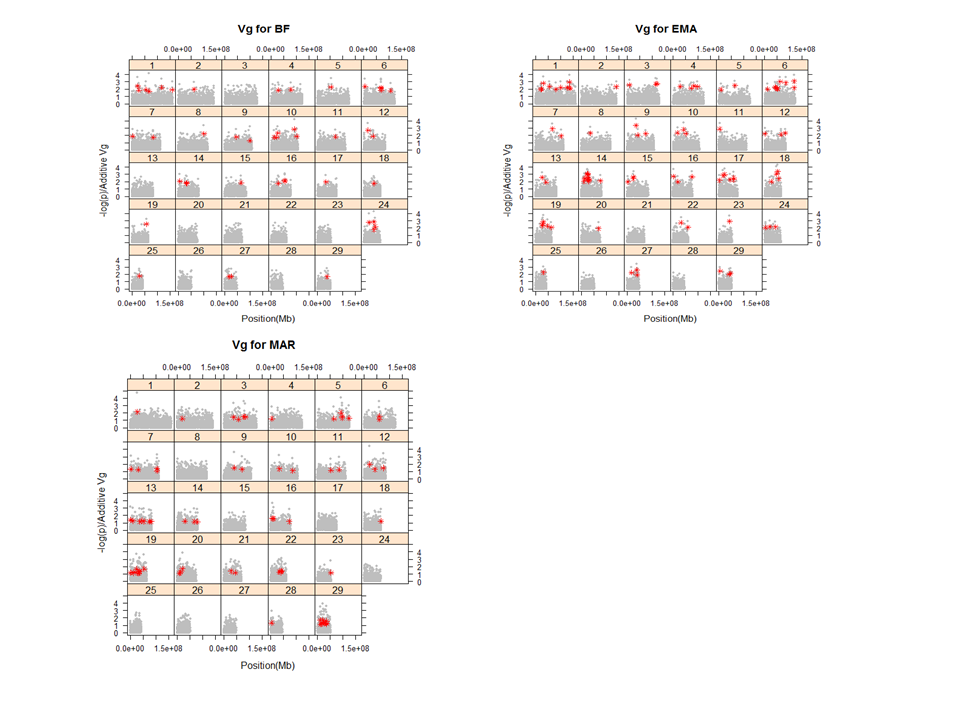

Supplement: Figure S2 — The -logP value for the association between SNP and carcass traits across genome (grey colour) and additive genetic variance (red colour) that significant SNPs (P<0.001) account for in single marker regression analysis. (A) Eye muscle area, (B) Back fat thickness and (C) Marbling score (MAR). (TIF) [file pone.0074677.s002.tif]

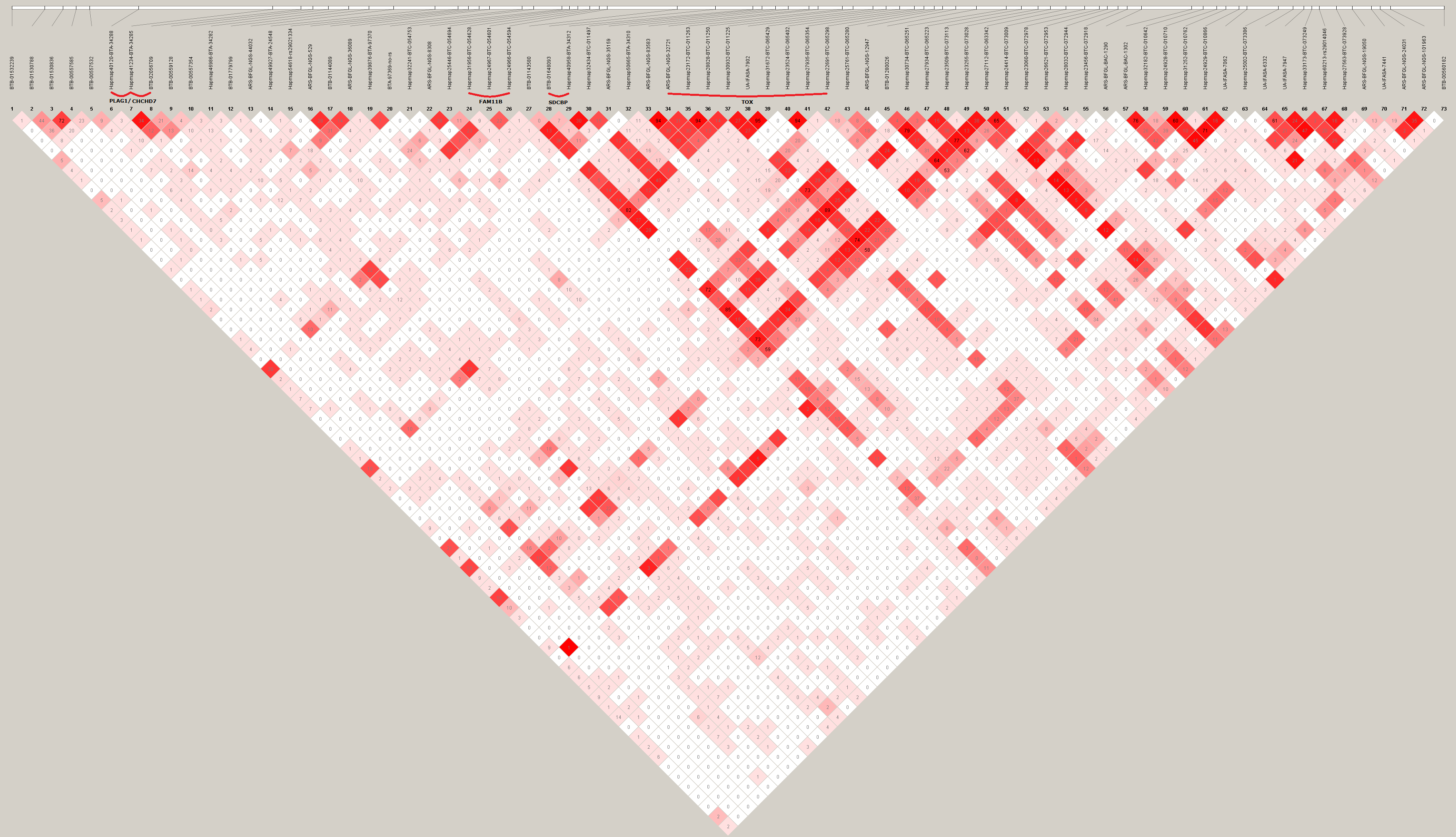

Supplement: Figure S3 — Linkage disequilibrium of the 29 SNPs surrounding 1.1 Mb BTA14 in Hanwoo. (PNG) [file pone.0074677.s003.png]

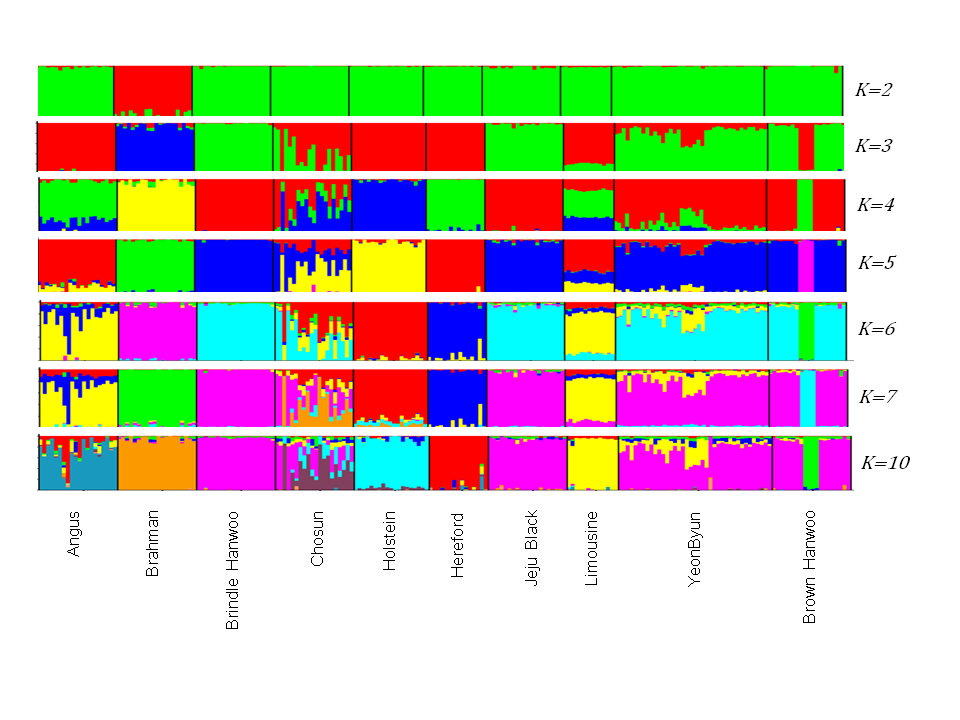

Supplement: Figure S4 — STRUCTURE result between Asian and European cattle breeds. (TIF) [file pone.0074677.s004.tif]
